# Diversity and correlation analysis of endophytes and metabolites of *Panax quinquefolius* L. in various tissues

**DOI:** 10.1186/s12870-023-04282-z

**Published:** 2023-05-25

**Authors:** Rui Li, Wanying Duan, Zhifang Ran, Xiaoli Chen, Hongxia Yu, Lei Fang, Lanping Guo, Jie Zhou

**Affiliations:** 1 School of Biological Science and Technology, University of Jinan, Jinan, 250022 PR China; 2 College of Pharmacy, Shandong University of Traditional Chinese Medicine, Jinan, 250355 PR China; 3 Weihai Wendeng District Dao-di Ginseng Industry Development Co. LTD, Weihai, 264407 PR China; 4 State Key Laboratory of Dao-di Herbs, National Resource Center for Chinese Medica, China Academy of Chinese Medical Sciences, Beijing, 100700 PR China

**Keywords:** *Panax quinquefolius* L, Endophytes, Various tissues, Diversity, Secondary metabolites, Correlation analysis

## Abstract

**Background:**

*Panax quinquefolius* L. (American ginseng) is widely used in medicine due to its wealth of diverse pharmacological effects. Endophytes colonize within *P. quinquefolius* in multiple tissue types. However, the relationship between endophytes and the production of their active ingredients in different parts of the plant is not clear.

**Results:**

In this study, the relationship of endophytic diversity and the metabolites produced in different plant tissues of *P. quinquefolius* were analyzed using metagenomic and metabolomic approaches. The results showed relatively similar endophyte composition in roots and fibrils, but obvious differences between endophyte populations in stems and leaves. Species abundance analysis showed that at the phylum level, the dominant bacterial phylum was Cyanobacteria for roots, fibrils, stems and leaves, Ascomycota forroots and fibrils roots, and Basidiomycota for stems and leaves. LC-MS/MS technology was used to quantitatively analyze the metabolites in different tissues of *P. quinquefolius*. A total of 398 metabolites and 294 differential metaboliteswere identified, mainly organic acids, sugars, amino acids, polyphenols, and saponins. Most of the differential metabolites were enriched in metabolic pathways such as phenylpropane biosynthesis, flavonoid biosynthesis, citric acid cycle, and amino acid biosynthesis. Correlation analysis showed a positive and negative correlation between the endophytes and the differential metabolites. Conexibacter significantly enriched in root and fibril was significantly positively correlated with saponin differential metabolites, while cyberlindnera significantly enriched in stem and leaf was significantly negatively correlated with differential metabolites (*p < 0.05*).

**Conclusion:**

The endophytic communities diversity were relatively similar in the roots and fibrils of *P. quinquefolius*, while there were greater differences between the stems and leaves. There was significant difference in metabolite content between different tissues of *P. quinquefolius*. Correlation analysis methods demonstrated a correlation between endophytes and differential metabolism.

**Supplementary Information:**

The online version contains supplementary material available at 10.1186/s12870-023-04282-z.

## Introduction

*Panax quinquefolius* L., also called American ginseng, is a remarkable plant with a history of medicinal use in China for over 300 years. *P. quinquefolius* has been shown to improve inflammatory processes, immune function, and response to exhaustion and stress [1–3]. Different tissues within *P. quinquefolius* contain various types of compounds with diverse pharmacological effects [4]. The global *P. quinquefolius* market reached US$ 85 million in 2018 [5], and China is now the world’s third largest producer of *P. quinquefolius* [6].The quality of American ginseng has been paid more and more attention [7].

Plant endophytes are microorganisms that exist in the intracellular and intercellular spaces of organs such as plant roots, stems, leaves and seeds, and generally do not cause diseases, but instead can form symbiotic relationships with the plant [8]. Many plant endophytes have important biological and ecological functions, such as promoting plant growth by fixing nitrogen, secreting auxin, resisting pests and diseases, or as potential biocontrol resources and carriers of exogenous genes as part of the complex community structure of plant endophytes [9, 10]. Plant roots, stems, leaves, flowers, seeds and other tissues represent their own unique microbial niches [11]. Highly diverse microbiota and significant variation of community structure were found in different tissues of rice [12]. For *Hevea brasiliensis* (rubber tree), there was substantial variation of the endophyte community composition among different plant organs [13]. For tomato, the diversity of endophytes differed in different tissues, with the highest diversity occurring in the roots [14]. Elucidating the variations of diversity and composition of plant tissues is essential for improving plant health and productivity. Although the diversity of endophytes in *P. quinquefolius* has been described [15], limited information is available on the endophytic community in different tissues of *P. quinquefolius*.

Highly-diverse endophytic communities can greatly influence the metabolite composition of host plants [16]. Studies on *Ginkgo biloba* L. found that endophytic bacteria were significantly correlated with flavonoid concentration and composition.In particular, *Staphylococcus* was positively correlated with quercetin and variations in the abundance of *Staphylococcus* showed a strong correlation with flavonoid content [17]. Gallic acid is the main active component of *Cynomorium songaricum* and concentration was significantly correlated with most of the dominant endophytic fungi [18]. Endophytes exist in the internal environment of the plant body and have co-evolved with the host plant, producing or participating in the synthesis of secondary metabolites similar to plant secondary metabolites [19, 20]. Thus, medicinal plant endophytes can affect the quality of Chinese herbal medicines.*P. quinquefolius* is rich in a variety of secondary metabolites and rich endophytic communities [14, 21]. In recent years 16s rDNA, ITS sequencing, PICRUSt and FUNGuild have been applied to comprehensively describe the composition, diversity and functional activity of plant endophytes, which provides a basis for studying the interaction between plant endophytes and host plants.Yet, there is scarce information available concerning the relationship between the diversity of endophytes and secondary metabolism in different tissues of *P. quinquefolius*. Therefore, it is of great value to study the relationship between endophytes and the production of active ingredients in different parts of *P. quinquefolius.*

In this study, the composition, diversity, and the predicted function of endophytes of *P. quinquefolius* in different tissues were explored by 16S and ITS2 rRNA sequencing techniques. Ultrahigh-performance liquid chromatography mass spectrometry (UPLC-MS) was carried out to explore the distribution of metabolites in four different tissues of *P. quinquefolius*. Pearson statistical method was used to analyze the possible correlation between endophytic bacteria and metabolites in *P. quinquefolius*. The above study is expected to lay a foundation for further understanding of endophytic bacteria and secondary metabolites in *P. quinquefolius*.

## Results

### Results of surface sterilization of *P. quinquefolius*

After a certain period of observation, no colonies were observed in PDA and NA medium, which indicated that surface sterilization was effective and could be used for the subsequent determination of endophytic bacteria of *P. quinquefolius*.

### Deep validation of endophyte sequencing sequences of *P. quinquefolius*

**Fig. 1:**
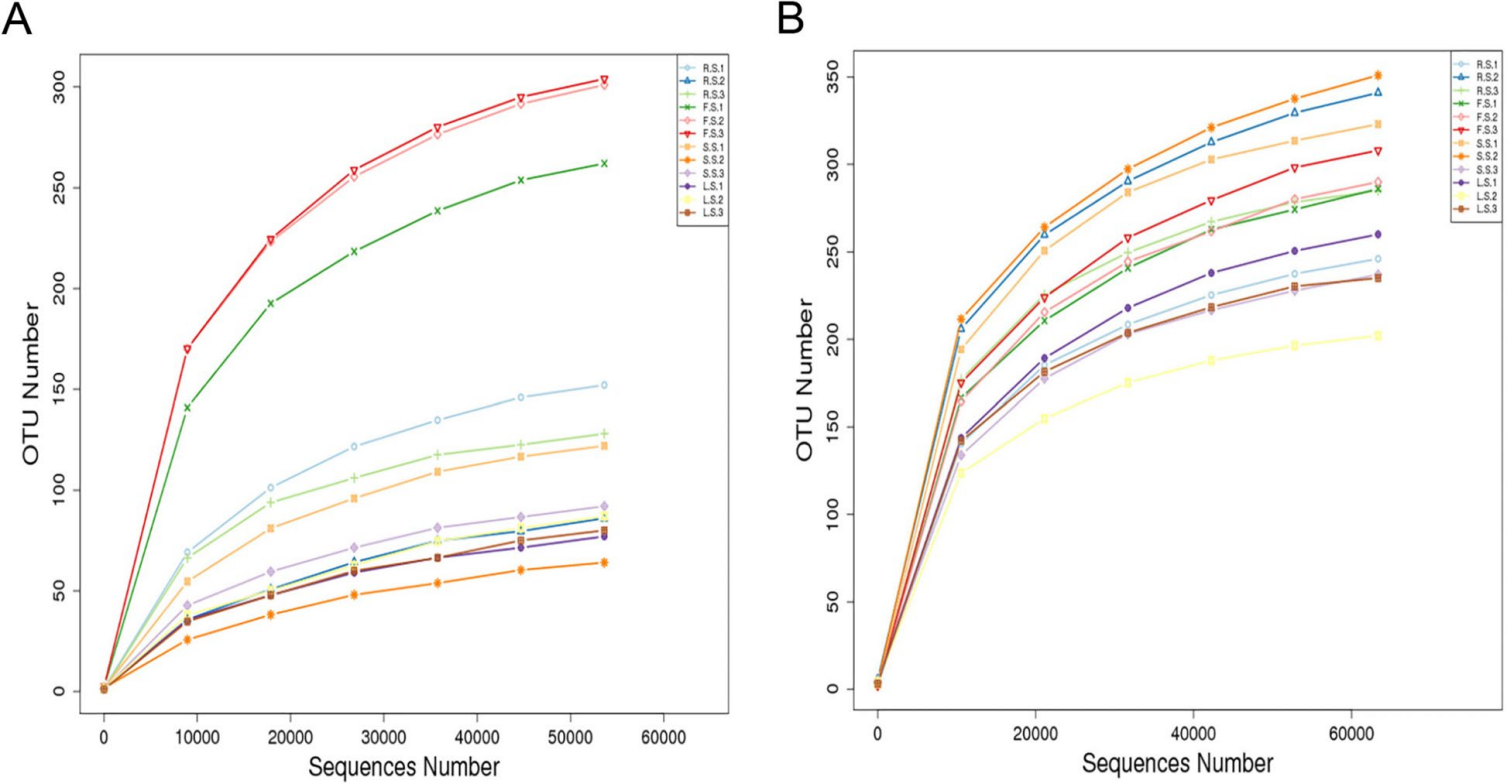
Rarefaction curves of endophytic bacteria (**A**) and fungi (**B**) in *P. quinquefolius* samples. (R.S.1, R.S.2, R.S.3) (F.S.1, F.S.2, F.S.3) (S.S.1, S.S.2, S.S.3)(L.S.1) (L.S.2) (L.S.3) are three repeats of root, fibril, stem and leaf respectively

A total of 1,797,737 high-quality sequences were obtained after quality control by high-throughput sequencing of samples from different tissues of *P. quinquefolius*. Among them, 866,196 were bacteria with an average length of 377 nt, which was consistent with the sequence length of the 16S rDNA V4 region. A total of 931,541 fungal sequences were obtained, with an average length ranging from 225 to 254 nt, which was consistent with the length of the ITS rDNA sequence. The rarefaction curve reflects the sampling depth of the sample and was used to assess whether the sequencing volume is sufficient to cover all taxa. The OTU dilution curves for each sample are shown in Fig. 1. The curves tend to be flat, and the OTU coverage rate of each production area is 99.9–100% (Table 1), indicating a reasonable amount of sequencing data for complete coverage. Thus, these data represent the endophyte community structure in the real environment with high confidence, indicating that we can effectively compare the endophyte communities in different tissues of the*P. quinquefolius* samples.

**Table 1:** Sequencing data and Alpha diversity index of sample ( *n, sx* = ± 3 )

Sample Name	Endophytic bacteria					Endophytic fungi				
	Effective Tags(#)	OTU	shannon	chao1	goods_coverage	Effective Tags(#)	OTU	shannon	chao1	goods_coverage
R.S.1	73,254	152	0.51	165.047	0.999	74,911	246	0.898	272.538	0.999
R.S.2	72,238	86	0.38	102.24	0.999	79,331	341	3.141	374.396	0.999
R.S.3	68,140	128	0.452	143.955	0.999	69,118	285	1.412	299.167	0.999
F.S.1	67,662	262	1.144	290.333	0.999	82,691	286	1.805	316.306	0.999
F.S.2	63,331	301	1.37	321.788	0.999	83,021	290	1.381	321.294	0.999
F.S.3	68,895	304	1.253	321.679	0.999	82,885	308	1.538	340.679	0.999
S.S.1	75,740	122	0.464	131.22	0.999	74,952	323	2.198	341.812	0.999
S.S.2	82,987	64	0.257	82.071	1	64,187	351	2.513	380.647	0.999
S.S.3	77,597	92	0.37	107	1	75,349	237	3.291	253.957	0.999
L.S.1	82,615	77	0.365	98.938	0.999	87,712	260	2.12	276.893	0.999
L.S.2	79,944	87	0.359	111	0.999	74,712	202	2.421	211.75	1
L.S.3	53,793	80	0.3	90.714	1	82,672	235	2.428	246.733	0.999

### Diversity of endophytes among *P. quinquefolius* tissues

**Fig. 2:**
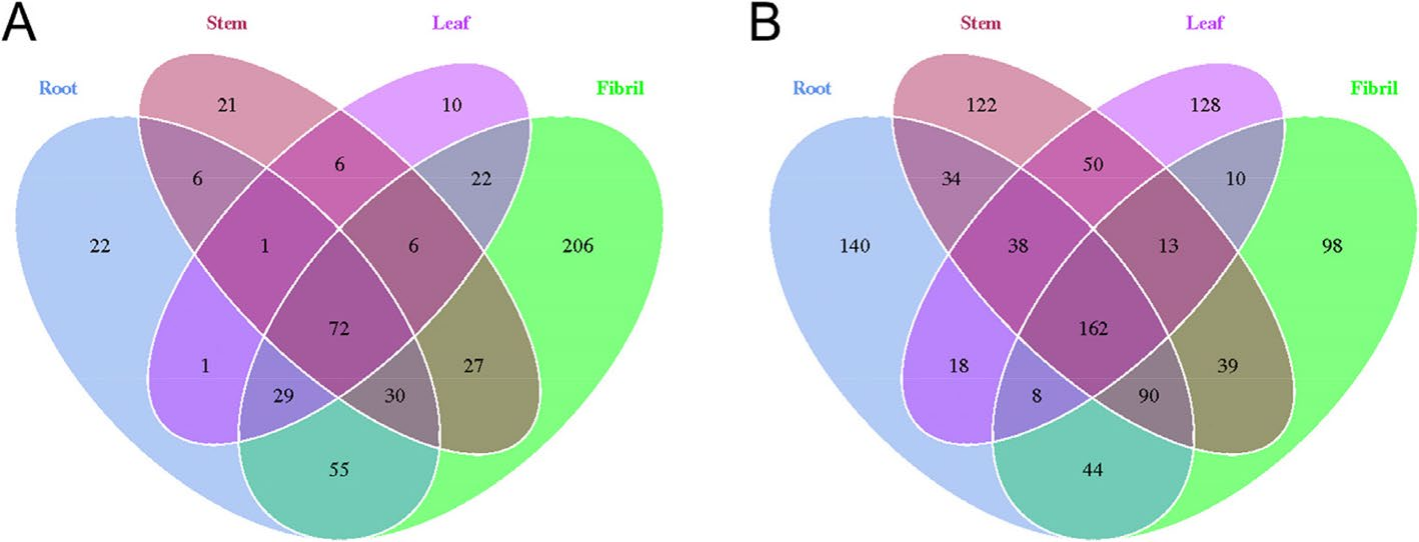
Venn diagram of OTU distribution of endophytic bacteria (**A**) and fungi (**B**) in different tissues of *P. quinquefolius*

**Fig. 3:**
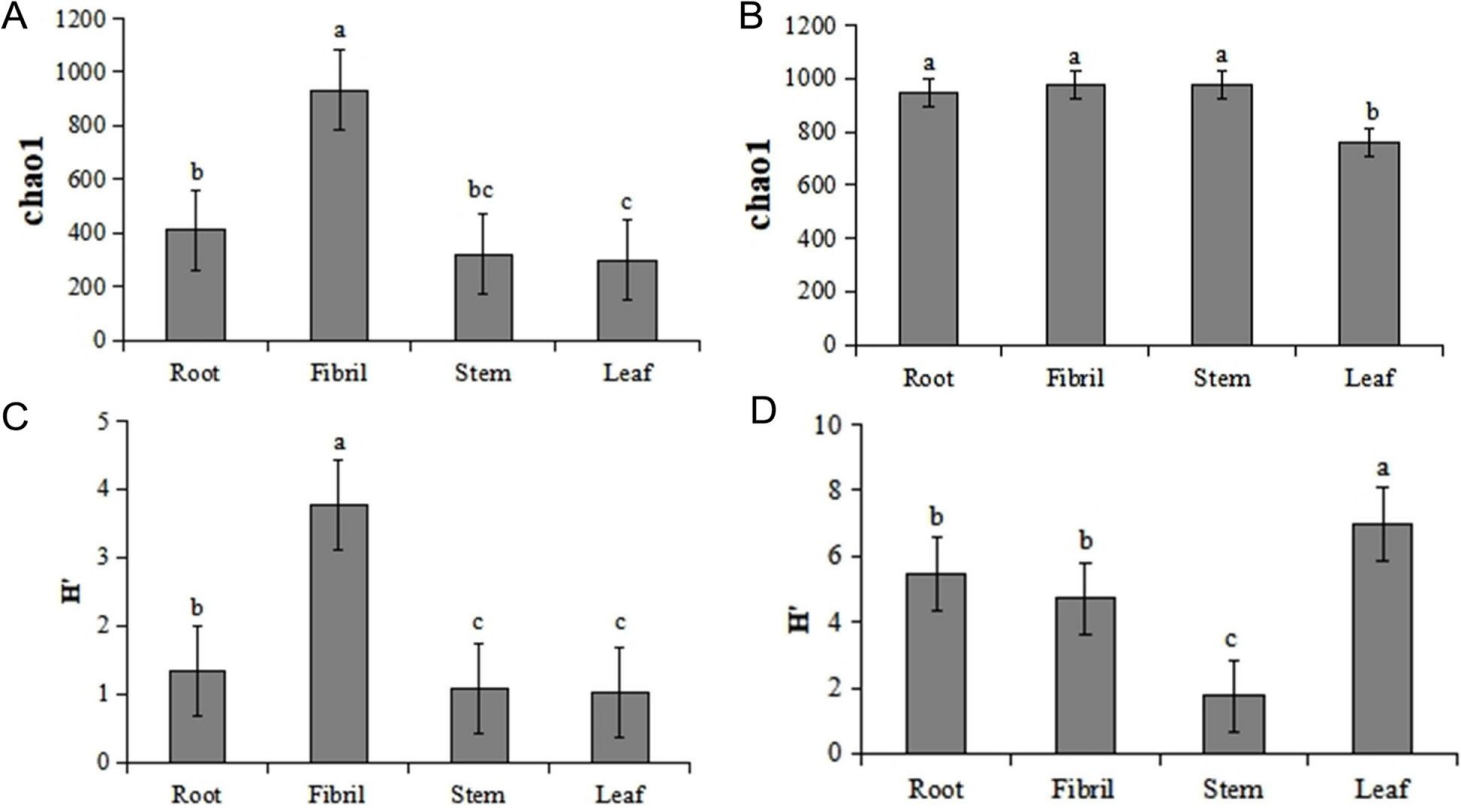
Changes of endophytic bacteria Chao 1 (**A**) and Shannon index (**C**) and fungi Chao 1 (**B**) and Shannon index (**D**) in *P. quinquefolius.* Each value represents the mean ± SD of *n* = 3. Different letters indicate significant differences at the 0.05 level

A Venn diagram was constructed at the OTU level to analyze the composition of the species contained within a sample. As shown in Fig. 2, the fibrils showed the highest number of bacterial OTUs, with the lowest number of OTUs in leaves. The highest fungal OTUs content was found in the roots, and the lowest fungal OTUs content was found in the leaves. There were relatively few common OTUs in the four tissues of *P. quinquefolius*, indicating substantial differences in the composition of endophytic bacteria in different tissues. Alpha diversity indices were applied to analyze the abundance and diversity of sample species. The Chao1 richness index was used as abundance-based richness estimators, whereas the Shannon (H’) indexes was used to quantitatively describe biodiversity on the basis of species richness. The Alpha diversity index (Chao1 and Shannon) of *P. quinquefolius* microbiota varied among different tissues (Fig. 3). In the endophytic bacterial community, Chao1 showed a greater number of species in the fibril samples, followed by the root samples, and lower numbers in the leaf and stem samples. The H’ results showed highest diversity in the fibril samples, with similar levels of diversity in the leaf and stem samples. For the endophytic fungal community, Chao 1 indicated a greater number of species in the fibril root and stem samples and fewer species in the leaf samples. H’ results showed that the highest diversity was found in the leaf samples, the lowest diversity was found in the stem samples, and similar diversity levels were found in the fiber and root samples.

**Fig. 4:**
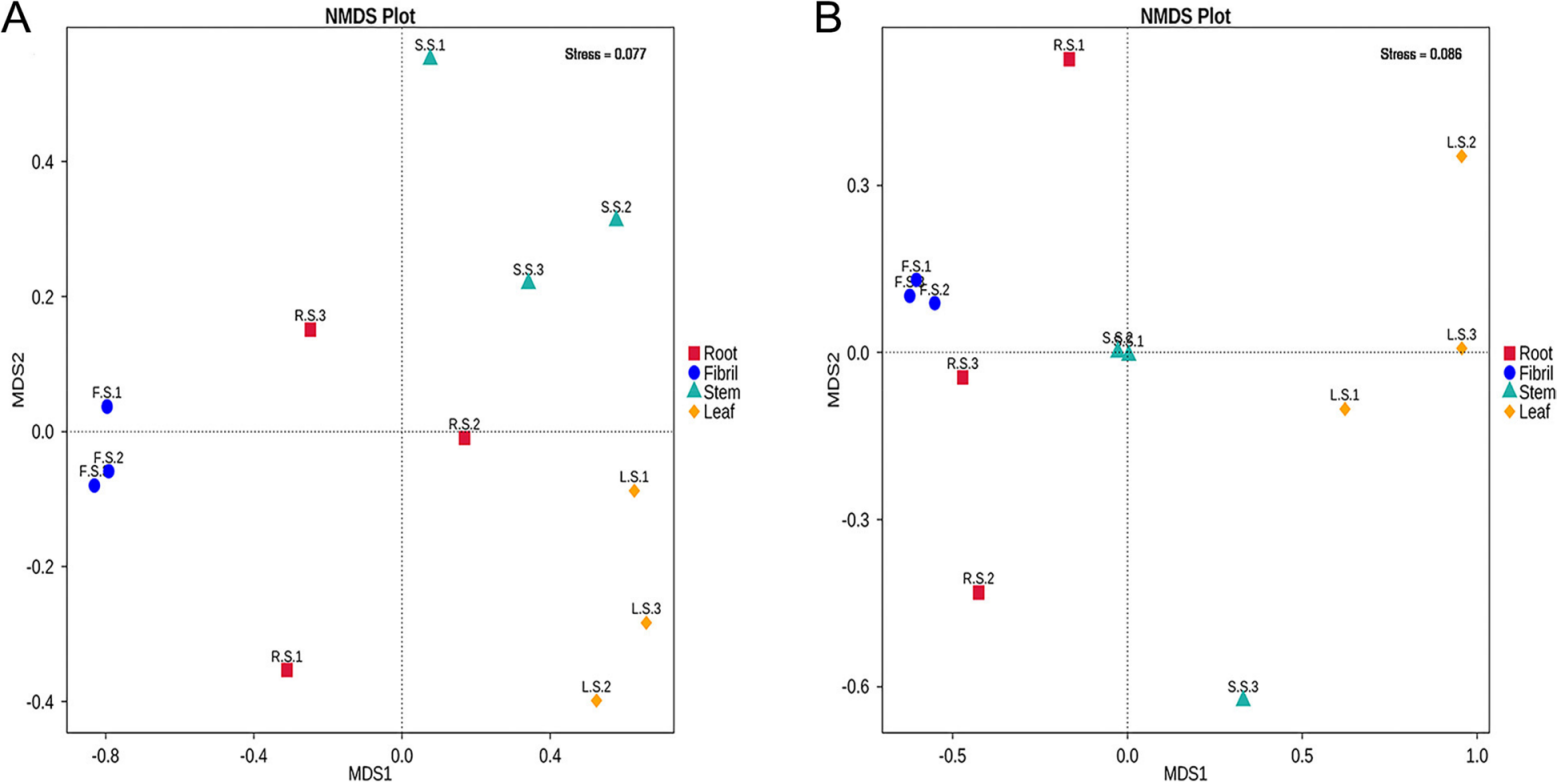
Multi-sample NMDS analysis of endophytic bacteria (**A**) and fungi (**B**) in *P. quinquefolius* samples. NMDS analysis, which each point in the diagram represents a sample, and samples from the same group are represented in the same color. The lower Stress (< 0.2) indicates that NMDS can accurately reflect the degree of difference between samples

Beta Diversity was assessed at the OTU level, and the composition of endophytic community structure in different parts was compared. NMDS analysis was applied to reflect the between- and within-group differences of the samples (Fig. 4). The analysis showed that the samples from each individual tissue of *P. quinquefolius* could be well separated, indicating significant differences in the endophytic communities in different tissues (*P* < 0.05).

### Composition of endophytes among different *P. quinquefolius* parts

**Fig. 5:**
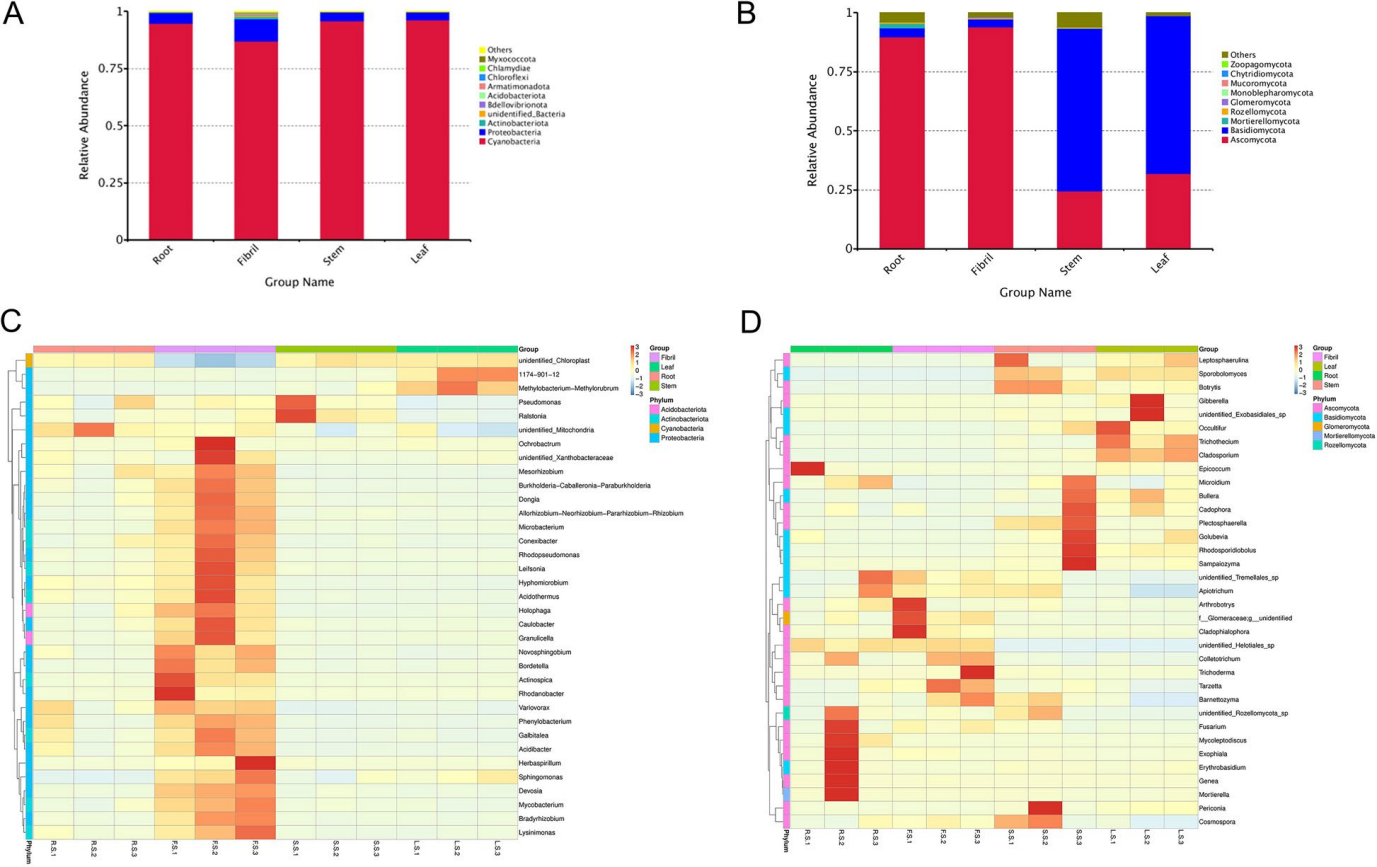
Relative abundance of endophytic bacteria (**A**) and fungi (**B**) in *P. quinquefolius* samples at the gate level, and heat map of endophytic bacteria (**C**) and fungal (**D**) communities

The reads from the 16S rRNA amplicon sequences detected from all samples mostly belonged to 23 different phyla containing a total 145 genera, with Cyanobacteria accounting for greater than 87% (Fig. 5A) of the amplicons. Heat map analysis of the relative abundance of endophytic bacteria at the genus level showed variation in samples from different tissues. In the fibril samples, the relative abundances of Bradyrhizobium, Rhodopseudomonas, Sphingomonas, Leifsonia, Acidibacter, and Rhodanobacter were significantly higher than those in other tissues (*P* < 0.05), and the relative abundance of unidentified_Chloroplast sequence in the fibril samples was significantly lower than that of the other samples. The relative abundance of Ralstonia in stem samples was significantly higher than that of other samples, and the relative abundance of unidentified_Mitochondria in root was significantly higher than that of the other samples (Fig. 5C).

Endophytic fungal communities in different niches were analyzed at the phylum, order, family, and genus level. A total of 11 phyla and 318 genera were detected. Ascomycota was identified as the dominant phylum of the root and fibril samples, with relative abundances of 89.80% and 94.05%, respectively. Basidiomycota was the dominant phylum in stem and leaf samples (68.85%, 66.81%, respectively) (Fig. 5B). Heat map analysis of the relative abundance of endophytic fungi at the genus level showed Barnettozyma, Cladophialophora, Genea, Apiotrichum, Mycoleptodiscus, Exophiala, Mortierella, Fusarium, unidentified_Helotiales_sp, Colletotrichum, Tarzetta, Paracremonium, Coprinellus, f__Glomeraceae, g__unidentified, unidentified_Tremellales_sp, unidentified_Rozellomycota_sp, Erythrobasidium, and Alternaria in root and fibril were significantly higher than those of stem and leaf samples, but Periconia, unidentified_Exobasidiales_sp, Occultifur, Rhodosporidiobolus, Sampaiozyma, Trichothecium, Sporobolomyces, Leptosphaerulina, Bullera, Golubevia, unidentified_ Sordariomycetes_sp, Plectosphaerella, Botrytis, Cadophora, Microidium, Cladosporium, Cosmospora and other genera were more abundant in stem and leaf samples compared to the abundances in the root and fibril samples (Fig. 5D).

### PICRUSt and FUNGuild functional prediction analysis

**Fig. 6:**
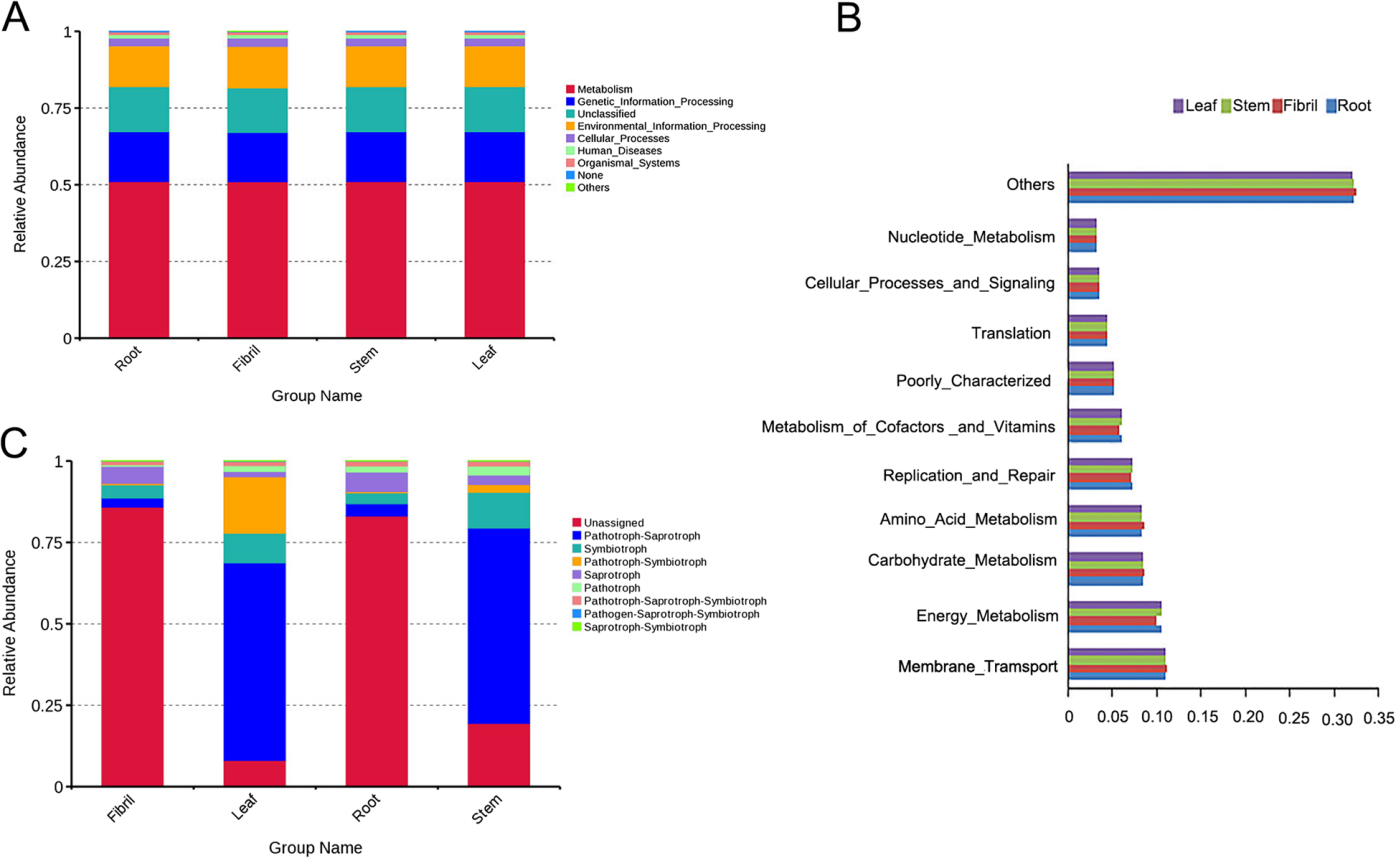
Functional annotation of the endogenous bacteria PICRUSt level 1(**A**), level 2(**B**) endogenous fungi FunGuild (**C**) of *P. quinquefolius* is a histogram of relative abundance

The endophytic bacteria OTU were compared with KEGG database, and the endophytic bacteria genes were predicted by PICRUSt. As shown in Fig. 6A, the main functions of endophytic bacteria in *P. quinquefolius* are metabolism, genetic information processing, cellular processes, environmental information processing, human diseases (Pathogenic bacteria, or the type of flora by which disease risk is assessed), and organ systems. The metabolism pathway was identified as the primary component in all samples, accounting for 51.0% (Fig. 6A). At KEGG Level 2, 10 metabolic pathways were identified, with energy metabolism, carbohydrate_metabolism, and metabolism_of_cofactors_and_vitamins accounting for a large proportion. The highest proportion was 25.4% for genes participating in energy_metabolism and carbohydrate_metabolism, and 25.3% in metabolism_of_cofactors_and_vitamins (Fig. 6B).

Fungal endophyte function of *P. quinquefolius* in different tissues predicted by FUNGuid is displayed in Fig. 6C. The results show that eight trophic mode groups could be classified: pathotroph-saprotroph, symbiotroph, pathotroph-symbiotroph, saprotroph, pathotroph, pathotroph-saprotroph-symbiotroph, pathogen-saprotroph-symbiotroph, and saprotroph-symbiotroph. OTUs that did not match any of the taxa in the database were classified as unassigned. Pathotroph-Saprotroph was the dominant trophic mode in leaf and stem samples, with relative abundances ranging from 47.14 to 47.70%, while symbiotroph was the dominant trophic mode in root and fibril samples (12.08% and 14.73%, respectively).

### Metabolomics studies in different parts of *P. quinquefolius*

**Fig. 7:**
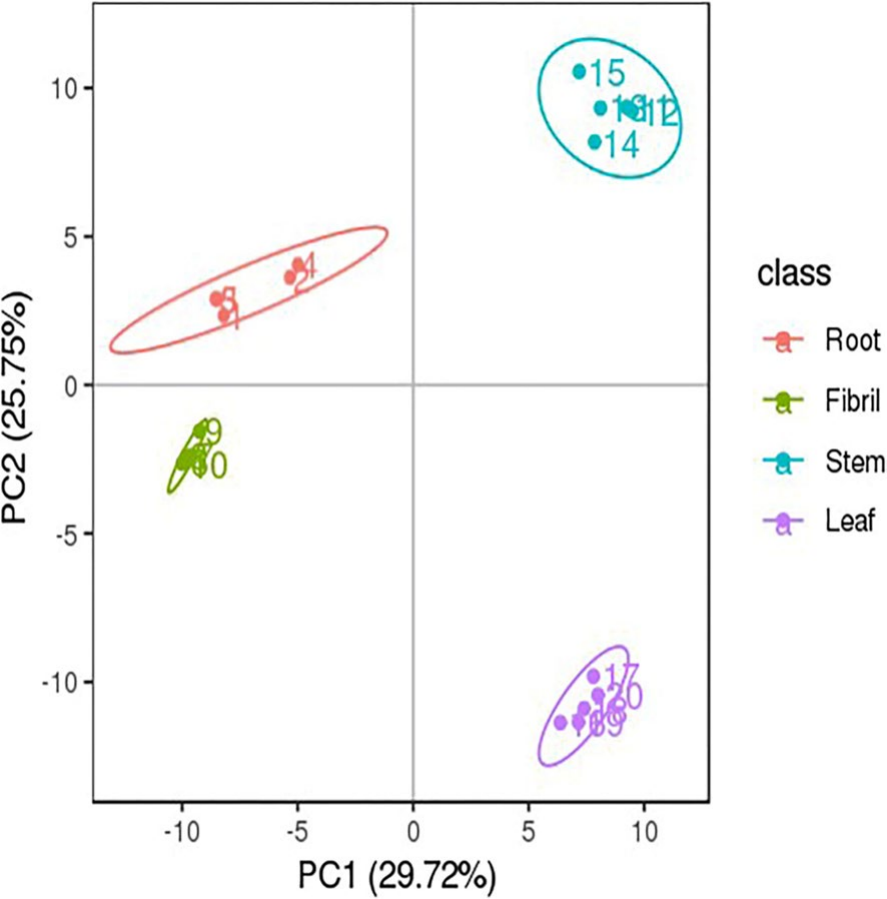
Multivariate statistical analysis of different tissues sample from *P. quinquefolius* PCA scores plot. Each point in the figure represents a sample, and samples from the same group are represented by the same color

**Fig. 8:**
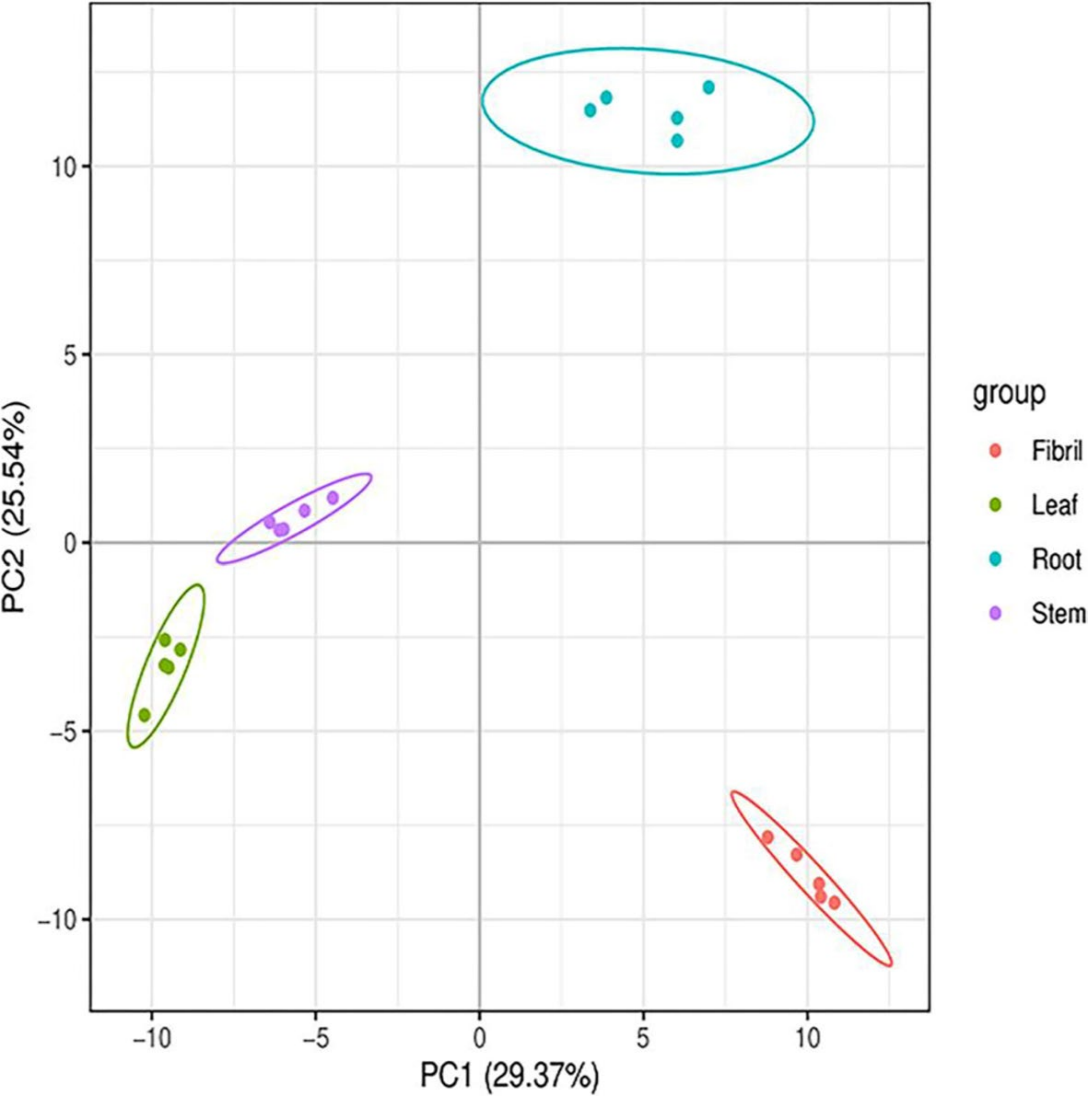
Multivariate statistical analysis of different tissues sample from *P. quinquefolius* PLS-DA scores plot. Each point in the figure represents a sample, and samples from the same group are represented by the same color

**Fig. 9:**
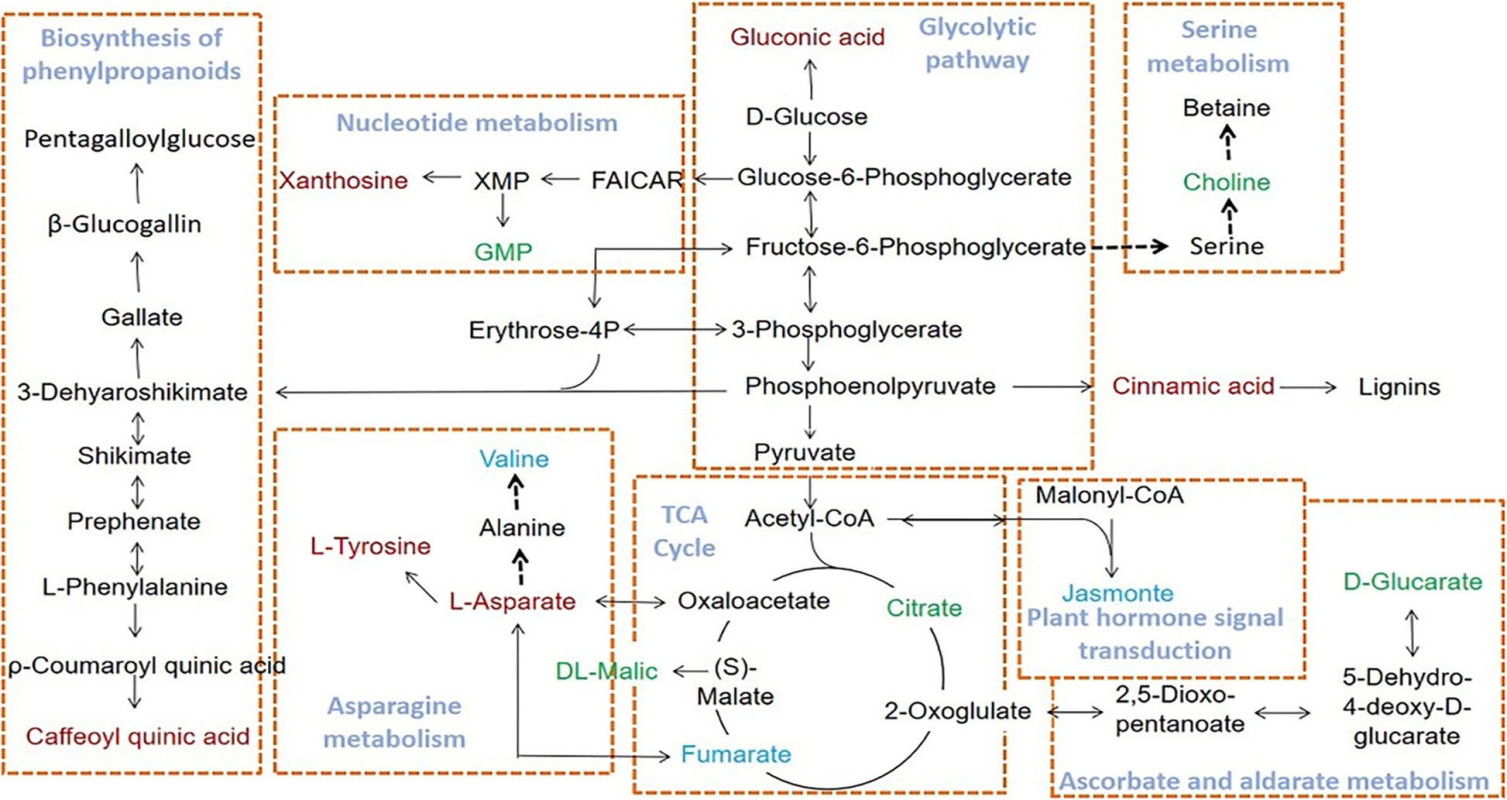
Study on the metabolic pathways of different metabolites of *p. quinquefolius*. Metabolites in blue featured higher relative contents in the Root, whereas metabolites in red presented higher relative contents in the Stem. Metabolites in greed featured higher relative contents in the Leaf. (https://www.kegg.jp/entry/map00020,https://www.kegg.jp/entry/map01061,https://www.kegg.jp/entry/map00010,https://www.kegg.jp/entry/map01232,https://www.kegg.jp/entry/map04075,https://www.kegg.jp/entry/map00053, https://www.kegg.jp/pathway/map00260)

**Fig. 10:**
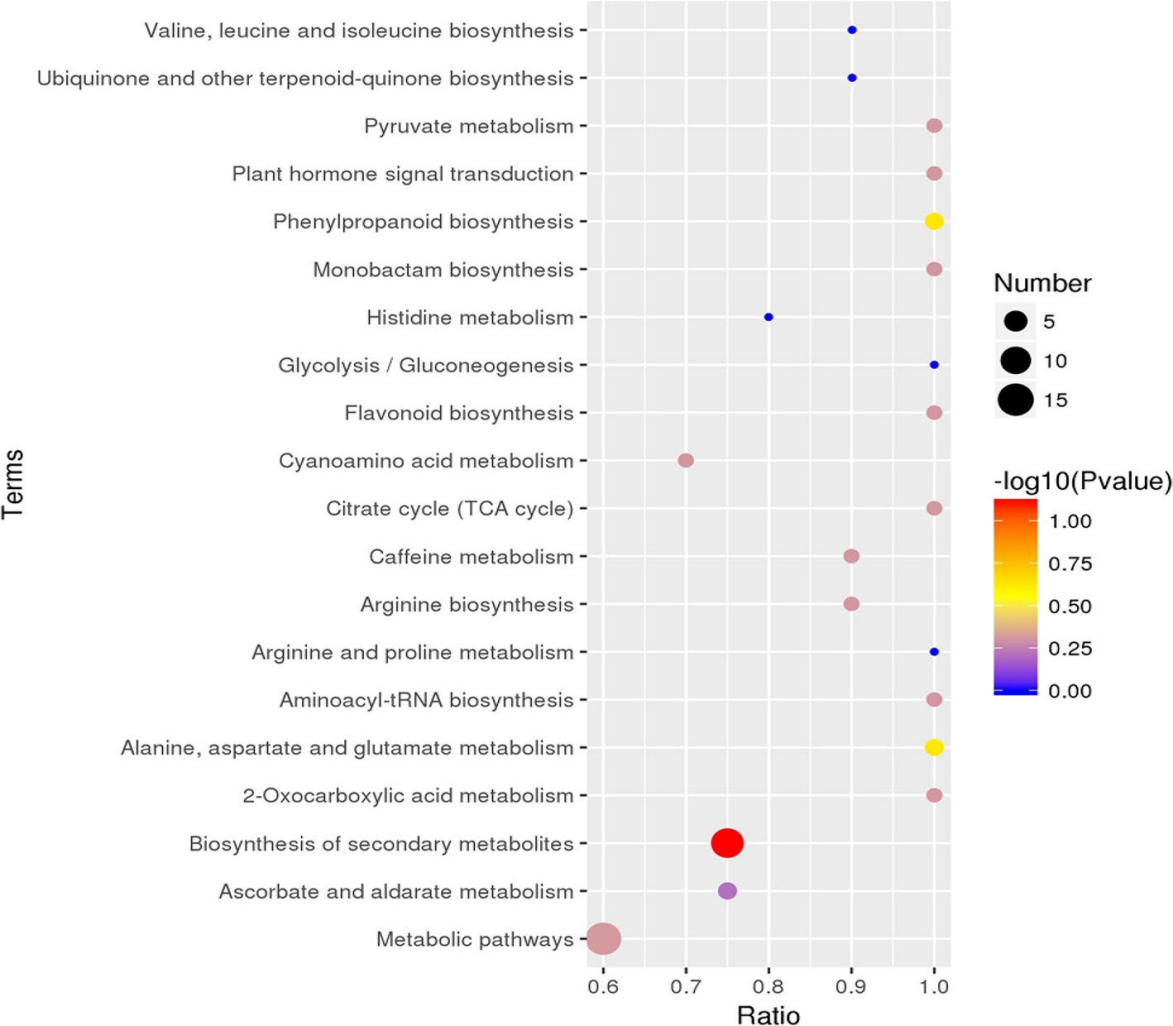
The top 20 KEGG pathway enrichment analysis. Each bubble represented a metabolic pathway whose abscissa and bubble size jointly indicated the influence factor of the pathway in topological analysis. The ordinate and bubble color indicated the *p* value of enrichment analysis which means the enrichment degree is more significant with the smaller *p* value

LC-MS/MS technology was used to quantitatively analyze the metabolites in different tissues of *P. quinquefolius*. A total of 398 metabolites were identified from all samples, including 229 positive ion mode (ESI+) metabolites and 169 negative ion mode (ESI-) metabolites (Table S1). The identified metabolites include organic acids, sugars, amino acids, polyphenols, and saponins. The expression data for all identified metabolites were analyzed using one-way ANOVA, with the samples compared in multiple groups, corrected by BH, and then compared for differential expression with *p*-value of 0.05 as the threshold, The obtained metabolites were classified by expression between samples and a total of 294 differential metabolites were found (Table S2). The 20 most differential metabolites are citric acid, DL-malic acid, α,α-trehalose, D-saccharic acid, gluconic acid, uridine 5’-diphosphogalactose, D-(-)-fructose, L-threonic acid, fumaric acid, guanosine monophosphate (GMP), L-tyrosine, 2’-deoxyinosine, xanthosine, L-aspartic acid, D-(-)-quinic acid, D-raffinose, chlorogenic acid, 2-isopropylmalic acid, jasmonic acid, and LPA 18:2. Due to the high dimensionality and high correlation of the metabolome data, we combined ANOVA with multivariate statistical analysis (PCA and PLS-DA) to systematically analyze the overall distribution trend between the: root, fibril, stem, and leaf samples. Each point in the PCA score plot represents a sample. As shown in the PCA score chart, the 20 samples can be clearly divided into four groups based on the tissue, with each group of samples more concentrated and clustered into a single category. This indicates that the metabolites in each group of samples are similar. However, there are differences in metabolic profiles between different groups, and this approach could be used to characterize chemical differences between the root, fibril, stem, and leaf (Fig. 7). The PLS-DA further revealed differences in chemical composition in the four plant parts (Fig. 8). Levels of citric acid, DL-malic acid, α,α-trehalose, D-saccharic acid, D-(-)-fructose, guanosine monophosphate (GMP), D-(-)-quinic acid, D-raffinose, 2-isopropylmalic acid were higher in the root samples, gluconic Acid, D-(-)-fructose, L-threonic acid, L-tyrosine, 2’-deoxyinosine, xanthosine, L-aspartic acid, chlorogenic acid were higher in stem samples, fumaric acid and jasmonic acid were higher in leaf samples, and LPA 18:2 was higher in fibril samples (Table S1). By searching the KEGG database (https://www.genome.jp/kegg/pathway.html) and referring to previous studies, a metabolic pathway involving the top 20 differential metabolites was constructed, clarifying the close relationship between the differential compounds in the metabolic spectrum of different parts of *P. quinquefolius *(Fig. 9). The top 20 KEGG pathways were identified. As shown, the significance was determined for each pathway by *p*-value and abundance factors, where metabolic pathways with larger bubbles and darker colors are the most significant (Fig. 10).

### Correlation analysis between endophytic diversity and metabolites of *P. quinquefolius*

**Fig. 11:**
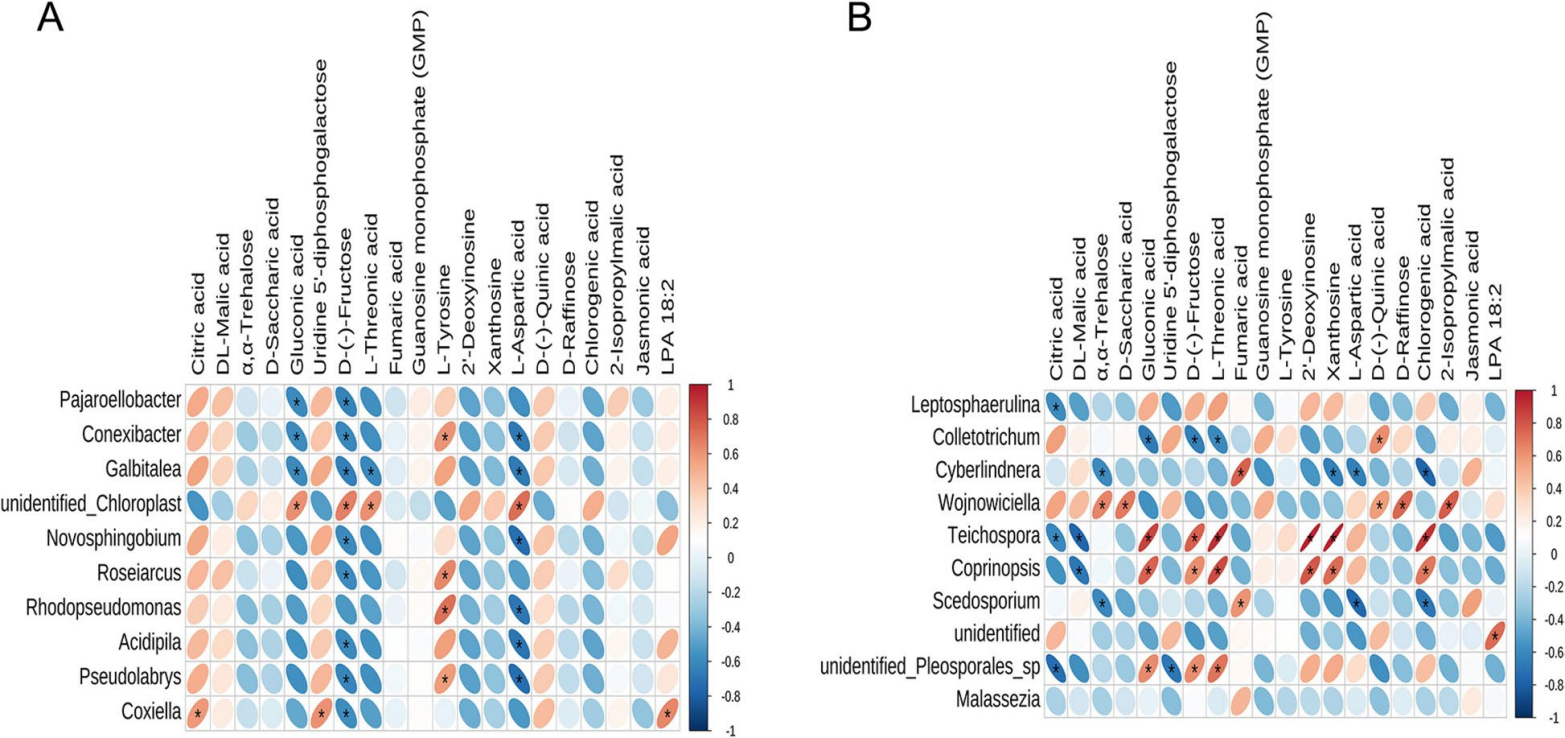
Heat map of the correlation between *P. quinquefolius* endophytic bacteria (**A**), fungi (**B**) and differential metabolites. The red and blue indicate positive and negative correlations. The color depth and circle size indicate the degree of correlation (*P < 0.05)

Correlation analysis was performed on the differential endophytes (top 10) and differential metabolites (top 20) of *P. quinquefolius* in different parts. It was obvious that 10 bacterial genera were significantly (*P < 0.05*) correlated with differential metabolites (Fig. 11A). Pajaroellobacter was negatively and significantly correlated with gluconic acid and D-(-)-fructose. Conexibacter was significantly negatively correlated with gluconic acid, D-(-)-fructose, and L-aspartic acid and positively correlated with L-tyrosine. Galbitalea was significantly and negatively correlated with gluconic acid, D-(-)-fructose, and L-threonic acid. Unidentified_Chloroplast showed a significant positive correlation with gluconic acid, D-(-)-fructose, L-threonic acid, and L-aspartic acid. Novosphingobium and Acidipila showed a significant negative correlation with D-(-)-fructose and L-aspartic acid. Roseiarcus showed a significant negative correlation with D-(-)-fructose and a significant positive correlation with L-tyrosine. Rhodopseudomonas showed a significant positive correlation with L-tyrosine and a significant negative correlation with L-aspartic acid. Pseudolabrys showed a significant negative correlation with D-(-)-fructose, L-aspartic acid and a significant positive correlation with L-tyrosine. Coxiella showed significant positive correlation with citric acid, uridine 5’-diphosphogalactose, LPA 18:2 and significant negative correlation with D-(-)-fructose.

As shown in Fig. 11B, a total of nine endophytic fungi (in the top 10 most abundant) were significantly (*P < 0.05*) related to differential metabolites of *P. quinquefolius* in different tissues. Leptosphaerulina was significantly correlated with citric acid only. Colletotrichum was significantly negatively correlated with gluconic acid, D-(-)-fructose and L-threonic acid, and significantly positively correlated with D-(-)-quinic acid. Cyberlindnera showed a significant negative correlation with α,α-trehalose, xanthosine, and L-aspartic acid and a significant positive correlation with fumaric acid. Wojnowiciella showed a significant positive correlation with α,α-trehalose, D-(-)-quinic acid, D-raffinose, and 2-isopropylmalic acid. Teichospora showed significant negative correlation with citric acid and DL-malic acid, and a significant positive correlation with gluconic acid, D-(-)-fructose, L-threonic acid, 2’-deoxyinosine, xanthosine, and 2-isopropylmalic acid. Coprinopsis showed a significant negative correlation with DL-malic acid and a significant positive correlation with gluconic acid, D-(-)-fructose, L-threonic acid, 2’-deoxyinosine, xanthosine, and chlorogenic acid. Scedosporium showed a significant negative correlation with α,α-trehalose, L-aspartic acid, chlorogenic acid and a significant negative correlation with fumaric acid. Unidentified_Pleosporales_sp showed a significant negative correlation with citric acid, uridine 5’-diphosphogalactose and a significant positive correlation with gluconic acid, D-(-)-fructose, L-threonic acid. Whether and how endophytes affect secondary metabolites needs further study.

**Fig. 12:**
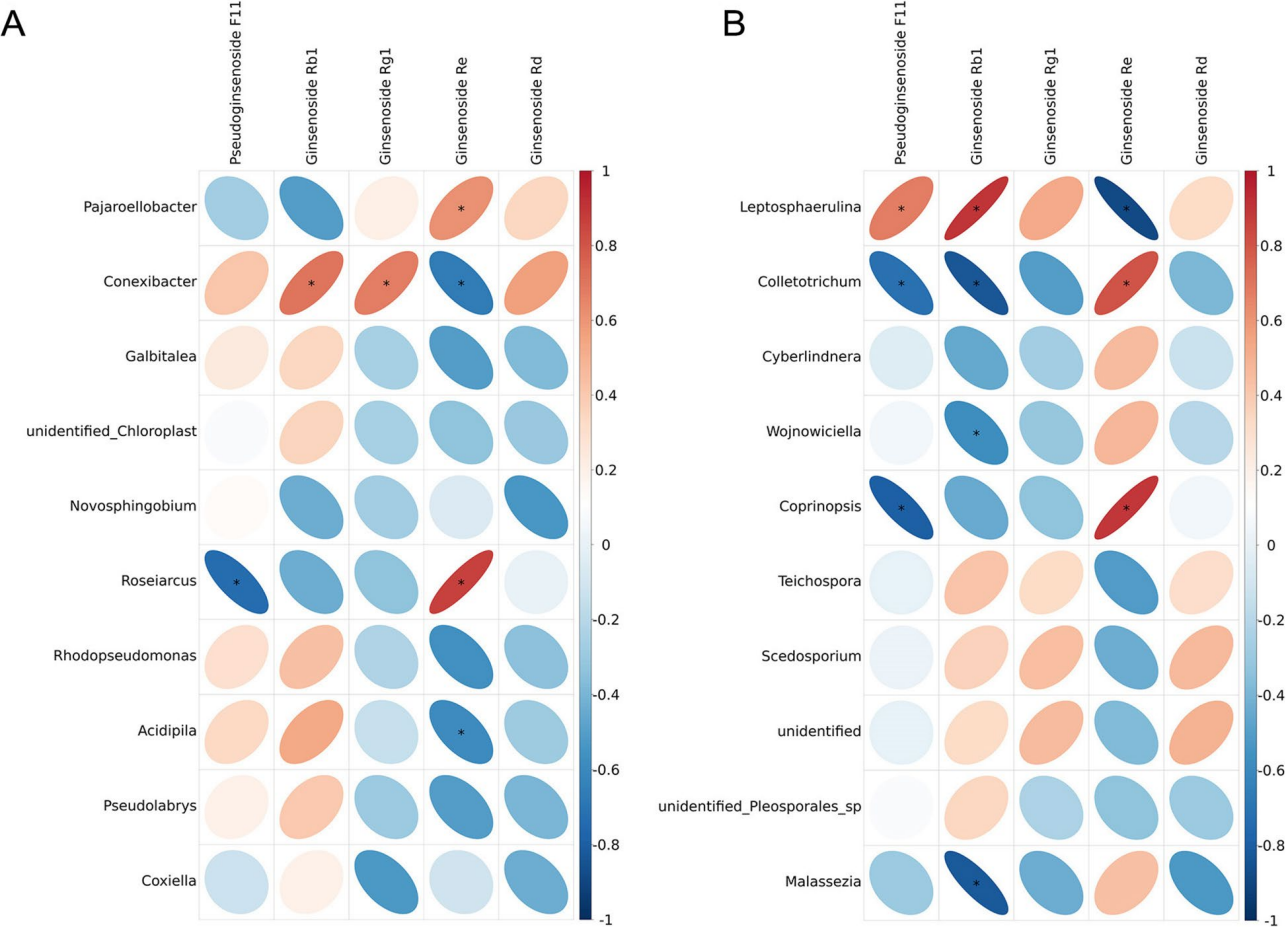
Heat map of the correlation between *P. quinquefolius* endophytic bacteria (**A**), fungi (**B**) and Saponins differential metabolites. The red and blue indicate positive and negative correlations. The color depth and circle size indicate the degree of correlation (*P < 0.05)

Correlations between saponin differential metabolites (Pseudoginsenoside F11, Ginsenoside Rb1, Ginsenoside Rg1, Ginsenoside Re, ginsenoside Rd) and the top 10 endophytes were analyzed. As shown in Fig. 12A, Pajaroellobacter showed significant positive correlation with Ginsenoside Re. Conexibacter was positively correlated with Ginsenoside Rb1 and Ginsenoside Rg1, and negatively correlated with ginsenoside Re. Roseiarcus showed a significant negative correlation with Pseudoginsenoside F11 and a significant positive correlation with Ginsenoside Re. Acidipila was significantly negatively correlated with Ginsenoside Re.

As shown in Fig. 12B, Leptosphaerulina is significantly positively correlated with Pseudoginsenoside F11, Ginsenoside Rb1, and significantly negatively correlated with Ginsenoside Re. Colletotrichum was significantly negatively correlated with Pseudoginsenoside F11, Ginsenoside Rb1, significantly positively correlated with Ginsenoside Re. Coprinopsis showed a significant negative correlation with Pseudoginsenoside F11 and a significant positive correlation with Ginsenoside Re. Malassezia had a significant negative correlation with Ginsenoside Rb1.

## Discussion

In our study, diversity of endophytes was correlated with different tissues of *P. quinquefolius*. Bacterial alpha diversity values were similar and generally higher in fibrils and roots compared to leaves and stems. The fungal alpha diversity values demonstrated that fibrils were more similar to roots than to leaves or stems. In previous similar studies, bacterial diversity was higher in *Populus* root samples than in leaf samples [22]. Likewise, the diversity of endophytic bacterial community of *Panax notoginseng* was similar in fibrils and roots [23]. In *Santiria apiculate*and *Rothmannia macrophylla*,endophytic diversity increased from leaves to roots [24]. Our NMDS analysis showed clear boundaries between the different tissues of *P. quinquefolius*, which is consistent with was reported for *Populus tremula* [25]. The results presented here demonstrate significant variation in the diversity of plant-associated microbial communities in different parts of the host plant.

Cyanobacteria, as a large phylum of bacteria, is the main contributor of biological nitrogen fixation in the ecosystem [26]. The roots, fibrils, stems and leaves of *P. quinquefolius* were enriched in Cyanobacteria, which may affect the growth of *P. quinquefolius*, and the biosynthesis of nitrogenous substance ssuch as alkaloids and proteins. This same result was reported in *Panax notoginseng* [27]. Ascomycota and Basidiomycota were the dominant fungal phylum found within *P. quinquefolius*,with Ascomycota dominant in fibrils and roots, and Basidiomycota dominant in leaves and stems. Ascomycota is the dominant phylum of fungal endophytes in many plants, including*Pinus thunbergii* and *Gentiana* [28, 29]. The relative abundances of bacterial endophytes, including Acidibacter, Bradyrhizobium, Rhodanobacte, and Ralstonia differed significantly among the four tissues of *P. quinquefolius*. In summary, beneficial flora may be selected by different parts of *P. quinquefolius*, creating a unique habitat conducive to healthy plant growth.PICRUSt analysis has been used to predict the function of endophytic bacteria in many plants [30, 31]. The functions of endophytic bacteria of *P. quinquefolius* mainly involve six aspects: metabolism, organic system, human disease, genetic information processing, environmental information processing, and cellular process. The number of genes related to metabolic process was the largest, accounting for 51.0% of the total. This is a similar result to that of Dawei Chen who examined the function of endophytic bacteria in *Rheum palmatum* [32]. In this study, the relative abundance of eight trophic pattern groups of endophytic fungi was different among different parts. This result is similar to that of Martínez-Diz et al. who used FUNGuild to study the functional groups of grapevine [33]. In this study, the functions of endophytic bacteria and fungi in the root, fibril, stem, and leaf of *P. quinquefolius* were analyzed, and the results provide a theoretical basis for future functional microbial strain isolation and excavation of endophytic bacteria in specific tissue types.

*P.quinquefolius* is rich in a variety of chemical components. Saponins are often used to evaluate the quality of *P.quinquefolius*, however other chemical components in addition to saponins have documented pharmacological effects [34, 35]. In our study, four tissues from *P. quinquefolius* plants were systematically analyzed and compared using untargeted metabolomics, revealing a diversity of metabolite forms. The variation of the distribution of metabolites in different tissues of *P. quinquefolius* was revealed by PCA and PLS-DA analysis, and the results were similar to the results in a previous study by Jiao Yufeng [36]. In these significant differential metabolites we selected, chlorogenic acid, which is more abundant in stems, and was reported to have important medicinal effects such as heat relief, detoxification, antibacterial and antiviral activity [37]. Dihydromyricetin exhibited anti-inflammatory, paroxysmal, hypotensive, and hypolipidemic effects [38]. Jasmonic acid is present at a high content in leaves and is often used as an endogenous growth regulator in higher plants [39]. Exploring the differences in metabolites in different plant tissues helps to identify the biochemical activities occurring in these tissues, which can promote the comprehensive utilization of *P. quinquefolius*.

Endophytes are selected for colonization by the plant environment, subsequently affecting the metabolism of host plants, resulting in a strong correlation of endophytes and metabolites [40]. In our study, differential metabolites were mostly positively correlated with the endophytic bacteria present in roots and negatively correlated with the endophytic bacteria in the stem and leaves, but the same correlations with differential metabolites did not occur with the endophytic fungi. In a previous study, the dominant bacteria in the root system of *Ephedra sinica* were positively correlated with differential metabolites, while the dominant bacteria in the stem were negatively correlated with differential metabolites, a result that was similar to our findings [40]. Additional studies have shown that E. sinica endophytic fungi and their communities directly influence the formation and accumulation of secondary metabolites, including bioactive substances [41]. In this study, differential metabolites involved in multiple biosynthetic pathways were significantly positively and negatively correlated with endophytes, and we speculate that endophytes influence the biosynthetic pathways of some metabolites. Endophytes in different parts of *P. quinquefolius* showed complex positive and negative correlation with metabolites, while whether there is a definite correlation between the two needs further study. In future studies, we plan to introduce related endophytes back to *P. quinquefolius* for further investigating the correlation between endophytes and secondary metabolism. If there is a correlation, the regulatory mechanism will be further studied.

## Conclusion

In summary, the endophytic communities diversity were relatively similar in the roots and fibrils of *P. quinquefolius*, while there were greater differences between the stems and leaves. As dominant endophytes, Cyanobacteria, Ascomycota and Bacteroidetes were abundant in various tissues of *P. quinquefolius*. There was significant difference in metabolite content between different tissues of *P. quinquefolius*. There was a positive and negative correlation between endophytes and differential metabolites, which provides an important basis for understanding the interaction between endophytes and metabolites.

## Methods

### Plant materials

**Fig. 13:**
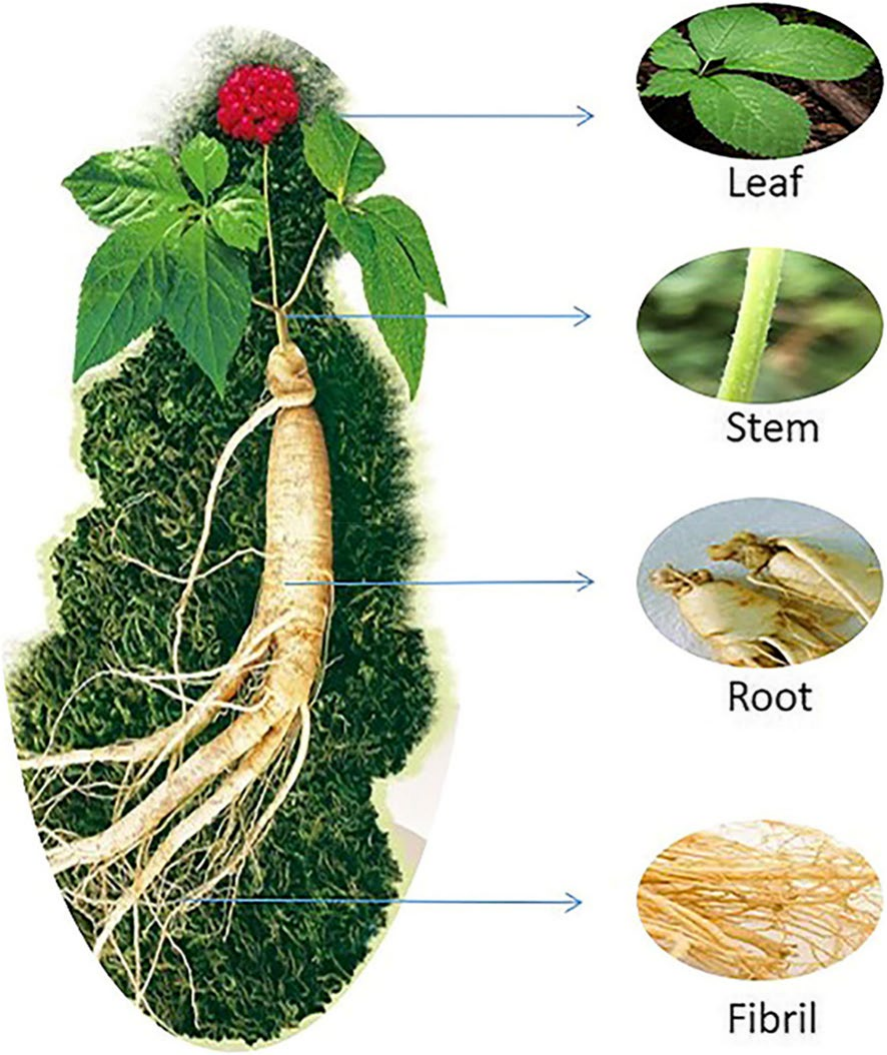
The sampling diagram of various tissues of *P. quinquefolius*

Four-year-old *P. quinquefolius* were collected from Wendeng, Shandong Province, China (37.25’N and 122.08’E), a main production district for *P. quinquefolius*. For the survey, three biological replicates were collected from five sites in the main production district for *P. quinquefolius*. Fifteen healthy *P. quinquefolius* plants were collected in August 2021 and pooled as a single biological replicate and separated into four tissue types (root, fibril, leaf and stem). The sampling diagram of various tissues of *P. quinquefolius* is shown in Fig. 13. Samples derived from the same strain were divided into two parts, with one part used for endophytic diversity analysis and one part was used for metabolomics analysis. The samples were identified by Prof. Lanping Guo and deposited in the School of Biological Sciences and Technology, University of Jinan (deposition number: 371003YC0024).

### 16S and ITS2 rRNA sequencing analysis of *P. quinquefolius* endophytes

To sterilize the surface of the plant from exogenous bacteria and fungi, all subsamples used for endophytic diversity analyses were immersed in 70% ethanol for 5 min, 2.5% NaClO solution for 1–2 min, 70% ethanol for 1 min, and then were rinsed five times in sterile water. To test for residual contamination, the supernatant from the last washing step was inoculated on PDA (potato dextrose agar) and incubated at 25 ℃ for 7 d and NA (nutrient agar) at 37 ℃ for 5 d respectively. All plant tissue samples were stored at − 80 ℃ until DNA extraction [42]. Total genomic DNA was extracted from all samples by using the MOBIO PowerSoil® Kit (MOBIO Laboratories, Inc., Carlsbad, CA, USA), according to the manufacturer’s instructions. DNA was quantified using NanoDrop spectrophotometer and kept at -20 ℃ for further PCR amplification. All PCR reactions were carried out in 30 µL reactions with 15 µL of Phusion® High-Fidelity PCR Master Mix (New England Biolabs), 0.2µM of forward and reverse primers, and about 10 ng template DNA. The bacterial 16S gene was amplified with primers 799F (5’-AACMGGATTAGATACCCKG-3’) and 1193R (5’-ACGTCATCCCCACCTTCC-3’). The following thermal cycle scheme was adopted: initial denaturation at 98 ℃ for 1 min, followed by 30 cycles of denaturation at 98 ℃ for 10 s, annealing at 50 ℃ for 30 s, and elongation at 72 ℃ for 30 s, concluding with a last step of 72℃ for 5 min. The fungal ITS genes were amplified using the primers ITS1-1 F-F (5’-CTTGGTCATTTAGAGGAAGTAA-3’) and ITS1-1 F-R (5’-GCTGCGTTCTTCATCGATGC-3’). Amplification was utilized under the following conditions: initial denaturation at 98 ℃ for 30 s, followed by 6 cycles of denaturation at 98 ℃ for 15 s, annealing at 50℃ for 30 s, decreasing 0.5 ℃ in each cycle. Next, 29 cycles were performed of extension at 72 ℃ for 30 s, denaturation at 98 ℃ for 15 s, annealing at 50 ℃ for 30 s, followed by extension at 72 ℃ for 30 s.The final extension was carried out at 72 ℃ for 2 min. PCR products was mixed in equidensity ratios. Then, mixtures of PCR products were purified with GeneJET Gel Extraction Kit (Thermo Scientific), and sequencing libraries were generated using Illumina TruSeq DNA PCR-Free Library Preparation Kit (Illumina,USA) following manufacturer’s recommendations and index codes. The library quality was assessed on the Qubit@ 2.0 Fluorometer (Thermo Scientific) and Agilent Bioanalyzer 2100 system. Finally, the library was sequenced on an Illumina NovaSeq platform and 250 bp paired-end reads were generated. All fastq files were submitted to National Center for Biotechnology Information (NCBI). Accession numbers were PRJNA865013 for bacteria and PRJNA865122 for fungi.

The data were processed utilizing the QIIME pipeline, and bacterial and fungal sequences were trimmed and assigned to each sample based on their barcodes. The Uparse software (Uparse v7.0.1001 http://www.drive5.com/uparse/) [23] was used to cluster all the Effective Tags of all samples. By default, the sequences clustered with 97% identity as OTUs. Species annotation analysis was performed using the Mothur method and SILVA138 (http://www.arb-silva.de/) [43]. The SSUrRNA database [44] (set threshold of 0.8 to 1) was used to obtain taxonomic information, and at each taxonomic level: kingdom, phylum, class, order, family, genus, and species the counts of the community composition of each sample, Observed-otus, Chao1, Shannon, Simpson, ACE, Goods-coverage, were calculated using Qiime software (version 1.9.1), dilution curves. Non-metric multidimensional scaling ordination (NMDS) analysis was performed to discover the taxonomic dissimilarity between different parts based on unweighted distance metrics. Beta diversity on both weighted and unweighted unifrac were calculated by QIIME software (Version 1.9.1). Metabolic and ecologically relevant functions were annotated by PICRUSt for the 16S rDNA OTU and FUNGuild for the ITS OTU.

### Metabolomics analysis

All subsamples for metabolomic analysis were carefully washed, cut into small pieces and ground into powder in liquid nitrogen. The metabolite extraction was performed as follows: 0.1 g of experimental samples were collected and mixed with 1.0 ml of pure methanol (0.1% formic acid) and vortexed for 10 s; the mixture was subjected to ultrasound treatment for 10 min, frozen at -20 ℃ for 1 h, and centrifuged at 10,000 rpm for 10 min. The upper layer was collected, filtered with a 0.22 μm filter, and injected into the UPLC column connected to an electrospray ionization-QTOF/MS device to detect metabolites (Waters, UK). The gradient consisted of 0.1% formic acid in water (A) and acetonitrile (B). Linear gradient settings are as follows: 0-2 min, 99 − 80% A; 2-3 min, 80 − 50% A; 3-7 min, 50 − 20% A; 7-7.5 min, 20 − 1% A; 7.5-9 min, 1% A; 9-9.1 min, 1–99% A; 9.1-10 min 99% A. The column temperature was 40 °C and the flow rate was 0.2 ml/min. The off-camera data (.raw) file was imported into CD 3.1 library search software for processing, to predict the molecular formula through molecular ion peaks and fragment ions, and to integrate with mzCloud (https://www.mzcloud.org/), mzVault and Masslist databases.

These metabolites were annotated using public databases, including the KEGG database, HMDB database, and LIPIDMaps database. Principal components analysis (PCA) and Partial least squares discriminant analysis (PLS-DA) were carried out using the metaX. The default criteria for differential metabolite screening are VIP > 1, *P*-value < 0.05 and FC ≥ 2 or FC ≤ 0.5. KEGG enrichment analysis of differentially accumulated metabolites was performed using KOBAS 2.0 software [45].

### Correlation analysis

Correlations between endophytes and metabolites in different parts of *P. quinquefolius* were assessed by univariate and multiple linear regression analyses, including simple correlation (Pearson correlation coefficient) and multiple correlation coefficient analyses using IBM SPSS Statistics 19.0 (Chicago, IL). Pearson statistical method was used to calculate the correlation coefficient RHO and *P* values between the relative abundance of each differential genus and the quantitative values of different differential metabolites at the genus level. The correlation analysis heat map and network map were drawn using R language corrplot and mixOmics packages.

## Supplementary Information

**Additional file 1: Table S1.** Total metabolites of positive and negative ion patterns identified from different parts of *Panax quinquefolius* L.

**Additional file 2: Table S2.** Different metabolites from different parts of *Panax quinquefolius* L.

## Data Availability

The sequencing data generated in the study are deposited to the NCBI SRA database under Bioproject No. PRJNA865013 and PRJNA865122.

## Abbreviations

*P. quinquefolius*: *Panax quinquefolius *L.
OTU: Operational taxonomic unit
UPLC-MS: Ultrahigh-performance liquid chromatography mass spectrometry
HMDB: Human Metabolome Database
KEGG: Kyoto Encyclopedia of Genes and Genomes
