# Correction: Diversity and correlation analysis of endophytes and metabolites of *Panax quinquefolius* L. in various tissues

**DOI:** 10.1186/s12870-023-04340-6

**Published:** 2023-06-27

**Authors:** Rui Li, Wanying Duan, Zhifang Ran, Xiaoli Chen, Hongxia Yu, Lei Fang, Lanping Guo, Jie Zhou

**Affiliations:** 1 School of Biological Science and Technology, University of Jinan, Jinan, 250022 PR China; 2 College of Pharmacy, Shandong University of Traditional Chinese Medicine, Jinan, 250355 PR China; 3 Weihai Wendeng District Dao-Di Ginseng Industry Development Co. LTD, Weihai, 264407 PR China; 4 State Key Laboratory of Dao-Di Herbs, National Resource Center for Chinese Medica, China Academy of Chinese Medical Sciences, Beijing, 100700 PR China

**Correction:** ***BMC Plant Biol*** **23, 275 (2023)**

**https://doi.org/10.1186/s12870-023-04282-z**

Following publication of the original article [1], an error was identified in Fig. 6, specifically Fig. 6C; the revised figure was missed during production process.

**Fig. 6:**
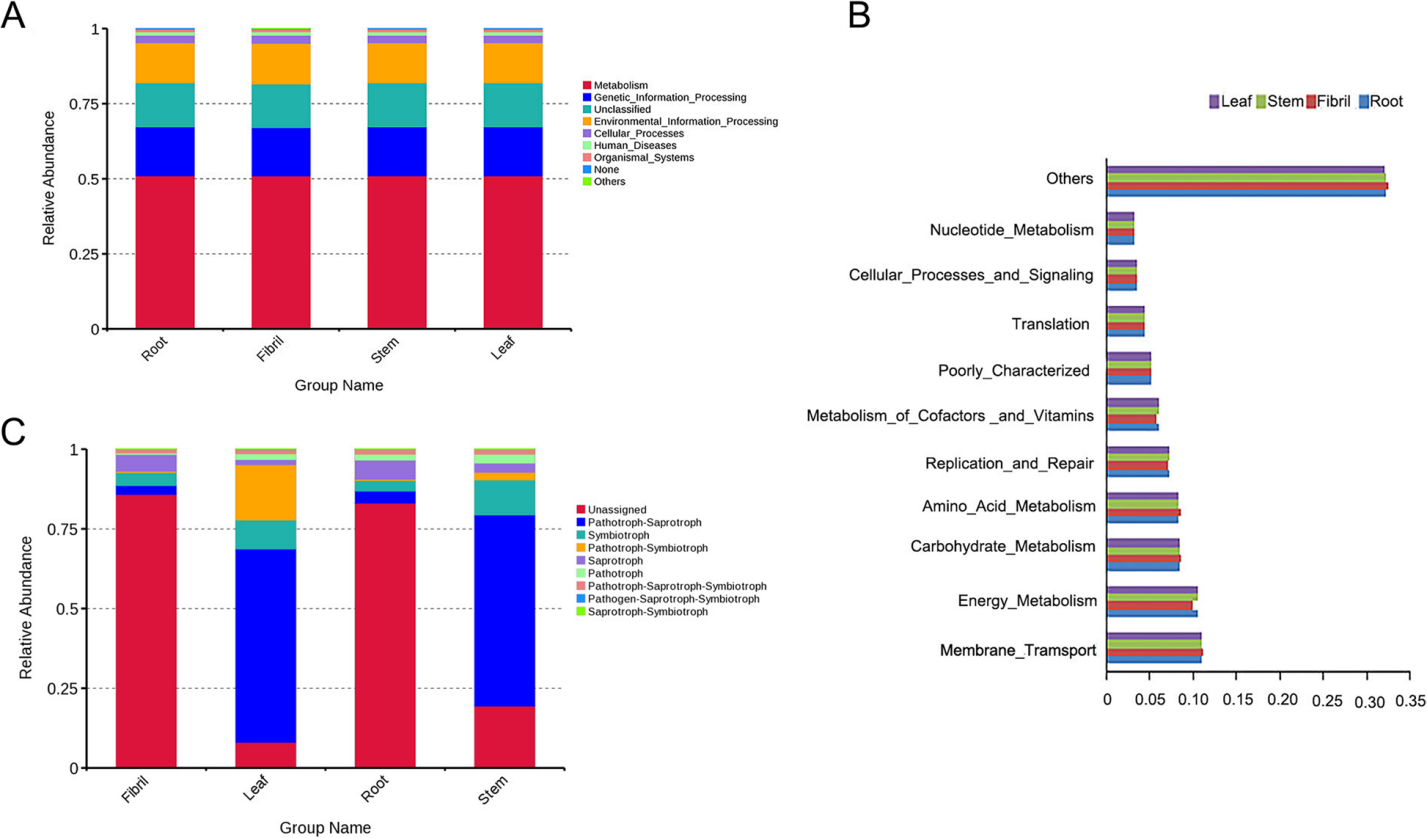
Functional annotation of the endogenous bacteria PICRUSt level 1 (**A**), level 2 (**B**) endogenous fungi FunGuild (**C**) of *P. quinquefolius* is a histogram of relative abundance

The correct figure is given below:

The original article [1] has been corrected.
